# Reference Values of Thyroid Hormones During the First Trimester of Pregnancy in Valencian Community (Spain) and Their Relationship with Iodine Intake

**DOI:** 10.3390/nu12051433

**Published:** 2020-05-15

**Authors:** María Teresa Murillo-Llorente, Carmen Fajardo-Montañana, Marcelino Pérez-Bermejo

**Affiliations:** 1 SONEV Research Group. School of Medicine and Health Sciences. Catholic University of Valencia San Vicente Mártir. C/Quevedo nº 2, 46001 Valencia, Spain; mt.murillo@ucv.es; 2 School of Medicine and Health Sciences. Catholic University of Valencia San Vicente Mártir. C/Quevedo nº 2, 46001 Valencia, Spain; carmen.fajardo@ucv.es; 3 Department of Endocrinology. Hospital Universitario de La Ribera. Carretera de Corbera, Km1. Alzira, 46600 Valencia, Spain

**Keywords:** reference values, thyroid hormones, ioduria, iodine deficiency, gestation, iodized salt

## Abstract

Thyroid hormones require special monitoring during the first trimester of gestation. Local reference values should be applied if available, especially in iodine-deficient areas, as generalized iodine supplementation is controversial. The aim of the present study was to establish thyroid stimulating hormone (TSH) and free thyroxine (FT4) reference values in the first trimester of gestation in the Valencian community (Spain) and relate them to iodine intake. A total of 261 healthy pregnant women participated in the study. The calculated reference values were 0.128–4.455 mIU/L for TSH and 0.9–1.592 ng/dL for FT4. The upper TSH reference value for pregnant women in the first trimester in our environment was similar to the latest American Thyroid Association (ATA) recommendation (4 mIU/L). The mean TSH value was significantly lower in smokers, and there were no significant differences when analyzing the influence of iodine supplementation, although the low duration of supplement intake needs to be taken into consideration. Ioduria levels (median 57 µg/L) confirmed iodine deficiency. We found statistically significant differences in ioduria levels among patients who consumed iodized salt and iodine supplements and those who did not. It is essential to focus on recommending adequate consumption of iodized salt and iodine supplements prior to gestation and at least during the first trimester to avoid possible maternal thyroid dysfunction and perinatal complications.

## 1. Introduction

Thyroid function during the first trimester of gestation is considered of clinical interest. The relationship between thyroid function and the consequences on fetal growth and psychomotor development in offspring has been well studied [1]. International medical literature states that between 5.7% and 11.8% of women will present thyroid dysfunction during pregnancy, so it is essential to maintain adequate concentrations of thyroid hormones to guarantee a correct psychomotor development of the fetus [2].

The need to establish thyroid screening with reference values according to each population area and adequate iodine supplementation continues to be a matter that requires further research, as recommended by the latest American Thyroid Association (ATA) Guidelines [3]. During pregnancy, thyroid stimulating hormone (TSH) values are lower than in the nonpregnant population due to the effect of chorionic gonadotropin, especially in the first trimester. In addition, during pregnancy, both the daily iodine requirements and the production of thyroxin (T4) and triiodothyronine (T3) are increased by 50%, which may trigger the development of hypothyroidism in advanced stages of pregnancy in women with iodine deficiency. This may lead to misclassification of women as euthyroid in the first trimester of gestation [4]. In 2007, different endocrine associations [5] stated that, in the absence of its own reference values, the upper limit of TSH during the first trimester of gestation should be 2.5 mIU/mL. Subsequently, in 2017, the ATA set the upper limit of normal TSH in the first trimester at 4.0 mIU/L while defending the need for specific reference values for TSH and free thyroxine (FT4) in each trimester of gestation [3]. These values may differ depending on the nutritional status of maternal iodine and the local laboratory analysis method (Table 1). 

Iodine intake needs to vary depending on the patient’s stage of life. It is known that iodine deficiency limits human development and learning capacity [14] and that our country produces foods with low iodine content [9]. Moreover, half of European inhabitants live in countries with iodine deficiency because the level of iodized salt distribution is diverse and there is a lack of government initiatives to ensure adequate iodine intake [2].

Iodized salt is the most appropriate medium of iodine supplementation because of its good risk-benefit ratio [15]. Its consumption is universal, and the quantities ingested are very similar throughout the year. Specifically, 3 g/day of iodized salt (one small teaspoon) covers the daily needs of this mineral [16,17]. However, the consumption of iodized salt is not enough to compensate the need for oligoelements in pregnant women, who need to take multivitamins with iodine to obtain adequate ioduria levels [18]. It is also known that the concentration of iodine in cow’s milk has increased in recent years, indicating that cow’s milk is a relevant source of iodine in our diet [19] that may be of clinical interest during gestation. 

**Figure 1:**
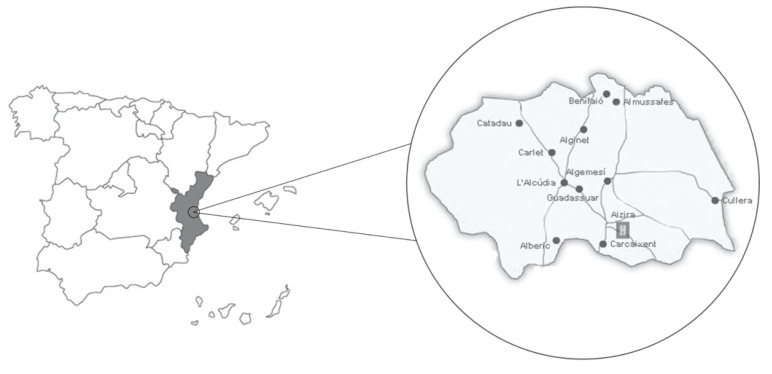
Study region. Source: own elaboration.

Figure 1 shows the area of Spain in which the study was carried out. In this region, as in the rest of Spain, iodized salt has, by law, an iodine concentration of 6 milligrams for every 100 grams of salt. Both whole and skimmed milk have an average iodine concentration of 9 micrograms/100 grams. In most of the studies performed in Spain (Table 1), TSH levels were similar, at their upper limit, to those established recently by the ATA [3], although there were notable differences in the analytical and statistical methods used.

We suspected that a TSH value < 2.5 mIU/L, suggested by ATA in 2007 as the universal reference value when local ones are not available [5], was too low for our pregnant women population at the first trimester as approximately 25% of our pregnant women had TSH ≥ 2.5 mIU/L in that period. To treat all of them could cause overtreatment and iatrogenesis. 

Furthermore, the status of iodine intake and ioduria levels were unknown. We also suspected that iodine was deficient in pregnant women in our area and that this had a relationship with TSH and FT4 results. Due to these problems, the aim of the present work was to establish our own reference values for TSH and FT4 in the first trimester of gestation in the Health Department of La Ribera (Valencia, Spain), as recommended by the American Thyroid Association since 2007 [3,5], and to relate them to iodized salt and iodine supplement intake. 

## 2. Materials and Methods 

### 2.1. Study Subjects

We carried out a cross-sectional study on 261 healthy women at the University Hospital of La Ribera at their first antenatal visit (gestational weeks 6–12) from September 2014 to January 2015. The study was approved by the Ethics and Clinical Research Committee of the University Hospital of La Ribera (accepted on 13 December 2013), and written informed consent was obtained from all participants. This work complies with the principles laid down in the Declaration of Helsinki.

The sampling had two stages. In the first stage, the health centers were chosen by means of a simple probabilistic sampling procedure from all the primary health centers in the health area of La Ribera. In the second stage, the pregnant women were selected successively until the estimated sample size was reached. Considering that there were 1591 deliveries at the University Hospital of La Ribera in 2013, for a 95% confidence interval (CI), a maximum expected error of 6%, and an expected proportion of 50%, we estimated a minimum sample size of 229 women. The collection of samples was carried out successively by a team of 18 trained midwives in the 10 participating health centers, and each center sample was proportional to the assigned population.

Pregnant women in the first trimester of pregnancy who were residents in the study area were included in the study. They were healthy and over 16 years old and presented no thyroid pathology at the time of inclusion.

Pregnant women undergoing treatment with drugs that influence and interfere with iodine metabolism (heparin, glucocorticoids, β-adrenergic blockers) and women who underwent thyroid hormone analysis in a laboratory other than that of the University Hospital of La Ribera were excluded. However, treatment with iron and aluminum hydroxide was not considered an exclusion criterion. 

### 2.2. Variables

The variables included in the study were sociodemographic (maternal age), obstetric (parity, gestational age), anthropometric (body mass index calculated at the first prenatal visit), iodine supplementation, iodized salt and milk intake, smoking habit, and analytical variables (TSH, FT4, and ioduria level before week 12 of gestation).

### 2.3. Laboratory Methods

TSH and FT4 determination were performed by TSH3-Ultra ADVIA Centaur and FT4 ADVIA Centaur XP assay (Siemens Healthcare Diagnostics, Munich, Germany). Values are expressed as mUI/L for TSH and ng/dL for FT4. Before this study, we only had local reference values for TSH (0.25–5.0 mIU/L) and FT4 (0.9–1.7 ng/dL) in the general population. 

For antithyroid peroxidase antibody (TPO Ab) determination, a solid-phase sequential enzymatic immunometric in vitro assay was performed by serum chemiluminescence, determined by an IMMULITE 2000 TPO Ab analyzer (Siemens Healthcare Diagnostics, Munich, Germany) and considering a normal value < 35 U/mL. Ioduria was performed using the Dunn colorimetric technique [20], and a desirable value > 150 μg/L was considered in pregnancy [3].

### 2.4. Data Analysis

Data were entered and stored in an MS Excel file and then transferred to SPSS v.23 software (SPSS Inc., Chicago, IL, USA) for statistical analysis. Normality of the data distribution was determined using the Kolmogorov–Smirnov test. Outliers were detected using the Reed criterion [21]. The same criterion applies for minimum values. The data were presented using the central CI of 95% (percentile 2.5 and 97.5) for TSH and FT4, mean and standard deviation (SD) in case of normal distribution or median, and interquartile range (IQR) if this was not the case. For the analysis of continuous variables, the comparison between the values was made by unpaired Student’s *t*-test in case of normality. The nonparametric Mann–Whitney test was used when the normality hypothesis was rejected when comparing two samples. The relationship between continuous variables was established using the Pearson’s correlation coefficient or the nonparametric Spearman’s correlation coefficient as needed. For the calculation of the reference values with nonparametric procedures, the 95% central interfractile interval was used. Two-sided *p* < 0.05 was considered statistically significant.

## 3. Results

Of the 275 pregnant women initially included in the study, 14 (5.1%) were excluded (two women with thyroid autoimmunity, one outlier, four cases of hypothyroidism, one case of hyperthyroidism, two abortions, one woman over 12 weeks of gestation, one woman aged under 16 years, one woman who did not undergo the analysis, and one woman who had provided analytical results from another institution). The final analyzed sample number was 261 women. Table 2 describes the sociodemographic and clinical characteristics of the sample.

A total of 246 women (94.25%) had not reached week 10 of gestation at the moment of sampling. The average TSH concentration was 1.9 ± 1.1 mIU/L (range 0.024–6.5 mIU/L), with 23.4% of the cases exhibiting TSH values **≥** 2.5 mIU/L. We did not observe a correlation between the TSH and FT4 hormones (*R* = −0.04, *p* > 0.05). Ioduria levels were < 100 μg/L in 74.71% of women and < 150 μg/L in 89.33%, and the values did not show any significant differences depending on week of gestation, BMI, number of previous pregnancies, ethnic group, or place of residence. 

Table 3 shows TSH, FT4, and ioduria levels, also taking into account the consumption of different types of salt and iodine supplements in pregnant women. We found statistically significant differences (*p* < 0.05) between the ioduria values of those that consumed iodized salt (65.4 μg/L, 37.6–100.6) and those who did not use it regularly (50.68 μg/L, 28.1–102.7) as well as between the group that consumed table salt (48.3 μg/L, 26.7–103.6) and the group that did not (61.1 μg/L, 36.7–101.6). The group that consumed iodized salt significantly increased their level of ioduria, and the group that consumed table salt also decreased it significantly.

A significant increase in ioduria levels (*p* < 0.05) was observed in those who took iodine supplements. A total of 184 women (70.5%) were taking iodine supplements. Of these, only 36 (19.6%) took it at daily doses of 150–200 micrograms in a pregestational manner, 81 (44%) started taking it after the recommendation of the midwife on their first visit, and 67 (36.4%) started taking it from the time they became aware of gestation. The duration of iodine supplementation had a median of 8.5 days (IQR = 26 days). No statistically significant differences were found in the hormonal levels (TSH, FT4) between those taking iodine supplements and those who did not.

We found statistically significant lower TSH levels in women who smoked compared to those who did not smoke prior to gestation and during the first trimester (1.51 ± 0.72 vs. 2.06 ± 1.13 mIU/L; *p* = 0.05). The mean self-reported tobacco use in the first trimester was 8.2 cigarettes per day (SD = 5.6).

We did not find statistically significant differences (*p* > 0.05) regarding FT4 and ioduria levels in smokers vs. nonsmokers or in those who took iodine supplements vs. those who did not.

The baseline values were calculated using the nonparametric method based on the range as the concentrations were not distributed in a Gaussian form. Table 4 shows the values of the central non-parametric interval of 95% of each analyzed hormone, limited by the 2.5 and 97.5 percentiles. 

## 4. Discussion

The need to determine the reference values for each population and laboratory is well known. Furthermore, there are differences in laboratory methods (type of test and units of measurement) and population characteristics (sample size, inclusion criteria, geographical area to study, and iodine sufficiency). Such differences often result in a wide range of reported results and can cause confusion in what should be considered a normal range. Early evaluation and treatment of thyroid dysfunction can prevent complications in pregnancy and childbirth [22,23,24].

During gestation, physiological changes occur that may contribute to misinterpretation of thyroid hormone values. Serum levels of TSH start to decrease significantly by the seventh week of gestation. Thyroid disorders are often common in young fertile women. It has been reported that between 5.7% and 11.8% of women will present thyroid dysfunction during pregnancy [2]. The prevalence of clinical gestational hypothyroidism is infrequent, and in the last decade, it has been established as being between 0.2% and 0.36%. Diagnosis of gestational thyroid disfunction is performed with FT4 and TSH determination as the assessment of total hormones is altered due to the high concentrations of thyroglobulin [25]. 

ATA considers that universal screening for thyroid disorder detection is not justified, although recommendation 97 indicates that it should be performed in iodine-deficient areas [3]. In this context, most of the studied women were in an iodine deficiency situation, taking into account the results of ioduria levels (median 57 μg/L (33.3–101.3). In fact, 74.71% were < 100 μg/L (minimum ioduria level recommended by the International Council for Control of Iodine Deficiency Disorders (ICCIDD)) [26] and 89.33% were < 150 μg/L (minimum ioduria level recommended by the World Health Organization (WHO) [3,5]. Although 70.5% were taking iodine supplements, the duration of the supplementation might be insufficient. 

The sample size is, in our judgement, sufficient and representative of the data to be conclusive, with the median age being 31 (16–45) years. In our study, we took into account the recommendations of different authors [4,26,27] regarding the exclusion of women with thyroid autoimmunity (TPO Ab > 35 U/mL) because of their association with increased concentrations of TSH, observing a slight change in the reference values that went from 0.127–4.471 mIU/L to 0.128–4.455 mIU/L. 

This upper normal value was significantly superior to the TSH < 2.5 mIU/L recommended by ATA in 2007 [5] and more similar to TSH < 4 mIU/L proposed in 2017 [3]. Our upper normal value (4.455 mIU/L) was just between the latest recommended by ATA (4 mIU/L) and our upper normal value for the general population (5 mIU/L). This new reference value started to be applied in June 2015 in our health department, avoiding treatment with levothyroxine (risking overtreatment and possible iatrogenesis) in 1 out of 4 pregnant women. 

We observed that our upper TSH limit value was higher when compared to national studies carried out in Cartagena [8], Aragón [6], and El Bierzo [11], although they were very similar to those observed in Cataluña [7], Jaen [9], Oviedo [10], Sevilla [12], and Vigo [13]. Most of the studies listed in Table 1 about thyroid hormone reference ranges were obtained from women at a gestational age of 7 to 12 weeks, agreeing with our results. The added value of our work that differentiates it from the previous ones lies in the inclusion of a concomitant iodine deficiency study. We also analyzed the relationship between ioduria and thyroid function (data not shown), finding that women with increased TSH are more likely to be iodine deficient. In the same way, the probability of being iodine deficient increases if the T4L is decreased. 

It is important to note that the lower limit value avoids the consideration of false subclinical hyperthyroidism in the first trimester if TSH is > 0.128 mIU/L, taking into account that our lower limit value for the general population is 0.27 mIU/L.

Some authors [28] have stated that TSH levels follow a circadian rhythm, with a peak during the night that drops during the morning, a circumstance that must be taken into consideration when performing blood extractions, as we did in our case.

Women consuming iodized salt, milk, and iodine supplements registered much higher ioduria medians than those who did not take them; in every case, our values were much lower than those recommended by the ICCIDD [26]. Therefore, as we found clear evidence of iodine deficiency in our population, we consider that it is not enough to simply recommend the consumption of iodized salt and milk. Furthermore, iodine supplementation is justified according to our local health authority recommendation (Conselleria de Sanitat Valenciana) [29]. A recent study showed that pregnant women who did not take iodine supplements showed lower values in neonatal parameters (gestational age, birth weight, and weight percentile) and a higher risk of prematurity [1], although, on the other hand, a recent systematic review did not find sufficient data to draw meaningful conclusions about the benefits and harms of routine iodine supplementation in women during pregnancy [30].

However, some authors [31] have countered the claim that iodized salt, milk, and their derivatives now allow the coverage of iodine needs during gestation. Milk is a fundamental food that can contribute, to a greater or lesser extent (depending on whether the animal is fed fodder or whether it grazes, its water intake, and disinfection of the udders), to dietary intake of iodine. A recent study [31] analyzed the iodine content of the most consumed milk in this country, concluding that one ration represents 20% (50 μg) of the recommended iodine intake in pregnant women. Furthermore, in this study, it was determined that there is no difference between the iodide content of whole milk, semi-skimmed milk, and skimmed milk. The effectiveness of iodized salt to prevent thyroid dysfunction is undoubted; however, the abuse of its consumption can lead to cardiovascular problems [32]. For this reason and because Spanish salt is one of the salts with the highest concentration of iodine in Europe, its consumption is recommended in moderation.

There are different methodological considerations to be taken into account in our study. The main limitation is not having performed an ultrasound scan to confirm the exact gestational age (it was calculated according to the last menstrual bleed). As a consequence, no reference values for each gestational week could be calculated for TSH and FT4, which could greatly increase the accuracy of diagnosis. By contrast, the strengths of our study are based on a probabilistic selection of primary care centers and the representativeness of the sample size. In addition, for the detection of outliers, the Reed criterion [21] and the fractile 2.5–97.5 [33] were used.

## 5. Conclusions

The upper TSH reference value for pregnant women in the first trimester in our environment (4.455 mIU/L) was similar to the latest ATA recommendation (4 mIU/L). Mean TSH value was significantly lower in smokers, and there were no significant differences when analyzing the influence of iodine supplementation, although the low duration of supplement intake needs to be taken into consideration. 

Having an iodine-deficient population (ioduria median of 57 µg/L), it is thus essential to focus on recommending adequate consumption of iodized salt, milk, and iodine supplements prior to gestation and at least during the first trimester of gestation in order to avoid possible maternal thyroid dysfunction and perinatal complications.

## Figures and Tables

**Table 1:** Different reference values of thyroid stimulating hormone (TSH) in Spain in the first trimester of gestation.

Area in Spain	TSH (mIU/L) P2.5 to P97.5^+^	*n* ^^^	Method
Aragón (Center) (Bocos-Terraz J et al., 2009 [6])	0.41–2.63 (WG ** 14)	330	Chemiluminescence immunoassay Abbott Diagnostics
Cataluña (Northeast) (Vila L et al., 2010 [7])	0.12–4.5 (WG 9)	178	Chemiluminescence immunoassay Advia Centaur Bayer
Cartagena (Southeast) (García de Guadiana L et al., 2010 [8])	0.047–3.466 (WG 11) 0.13–3.706 (WG 12)	117 151	Chemiluminescence immunoassay Roche Diagnostics
Jaén (Southeast) (Santiago P et al., 2011 [9])	0.23–4.18 (WG 7–10)	279	Chemiluminescence immunoassay Beckman Access
Oviedo, Asturias (North) (Aller Granda et al, 2013 [10])	0.17–4.15 (WG 6–12)	264	Chemiluminescence immunoassay Roche Diagnostic
El Bierzo (North) (Grifol ML et al., 2013 [11])	0.497–3.595 (WG < 12)	412	Chemiluminescence immunoassay ADVIA Centaur XP
Sevilla (Southwest) (Santiago P et al., 2015 [12])	0.36–4.49 (WG NI *)	NI *	Electrochemiluminescence immunoassay
Vigo (Northwest) (Pombar-Pérez et al., 2013 [13])	0.33–4.59 (WG < 12)	2410	Electrochemiluminescent immunometric analysis

* Not informed, ** week of gestation, +p: percentile, ^*n*: number of pregnant women.

**Table 2:** Sociodemographic characteristics of the sample.

.	Mean ± SD or *n* (%)	*p*-Value
Maternal age (years)	30.8 ± 5.1	
BMI first trimester (kg/m^2^)	23.9 ± 4.4	
Milk consumption		
No	36 (13.8%)	<0.001 *
Yes	225 (86.21%)	
Use of iodine supplements		
No	77 (29.5%)	<0.001 *
Yes	184 (70.5%)	
Use of iodized salt		
No	133 (51%)	0.757 *
Yes	128 (49%)	
Use of table salt		
No	166 (63.6%)	<0.001 *
Yes	95 (36.4%)	
Use of sea salt		
No	210 (80.5%)	<0.001 *
Yes	51 (19.5%)	
Smoking		
No	152 (58.24%)	0.008 *
Yes	109 (41.76%)	

BMI: body mass index, * chi-squared test.

**Table 3:** Clinical variables before week 12 of gestation.

	All	YES	NO	*p*-Value
TSH (mIU/L)	1.90 ± 1.05			
Milk consumption		1.92 ± 1.03	1.75 ± 1.13	0.39 *
Iodine supplements		1.94 ± 1.05	1.78 ± 1.05	0.25 *
Use of iodized salt		1.98 ± 1.06	1.81 ± 1.03	0.21 *
Use of table salt		1.64 ± 0.82	2.04 ± 1.13	0.003 *
Use of sea salt		2.03 ± 1.18	1.86 ± 1.01	0.29 *
Smoking		1.51 ± 0.72	2.06 ± 1.13	0.05 *
FT4 (ng/dL)	1.19 ± 0.20			
Milk consumption		1.19 ± 0.20	1.19 ± 0.16	0.95 *
Iodine supplements		1.19 ± 0.21	1.19 ± 0.17	0.94 *
Use of iodized salt		1.19 ± 0.21	1.19 ± 0.18	0.96 *
Use of table salt		1.17 ± 0.18	1.20 ± 0.21	0.40 *
Use of sea salt		1.22 ± 0.25	1.19 ± 0.18	0.27 *
Smoking		1.11 ± 0.30	1.18 ± 0.25	0.14 *
Ioduria (μg/L)	57 (33.3–101.3)			
Milk consumption		58.2 (33.5–103.3)	51.7 (30.2–98.4)	0.68 **
Iodine supplements		69.6 (44.9–109.9)	37.2 (25.6–80.5)	<0.001 **
Use of iodized salt		65.4 (37.6–100.6)	50.7 (28.1–102.7)	0.03 **
Use of table salt		48.3 (26.7–103.6)	61.1 (36.7–101.6)	0.03 **
Use of sea salt		51.6 (30.9–102.7)	59.0 (33.8–100.6)	0.36 **
Smoking		63.0 (39.2–109.7)	51.7 (29.1–100.6)	0.37 **

TSH and FT4 values are expressed as mean ± SD. Ioduria values are expressed as median (interquartile range). * Unpaired Student’s *t*-test; ** Mann–Whitney test.

**Table 4:** Reference interval TSH and FT4 (non-parametric method).

Confidence Interval 95%	Lower Value P2.5	Upper Value P97.5	Lower Limit P2.5	Lower Limit P97.5	Upper Limit P2.5	Upper Limit P97.5
General						
TSH	0.128	4.455	0.054	4.272	0.310	4.637
FT4	0.9	1.56	0.87	1.528	0.935	1.592
Use of iodized salt						
TSH						
No	0.10	4.13	0.026	3.947	0.282	4.312
Yes	0.18	4.70	0.106	4.517	0.362	4.883
FT4						
No	0.90	1.60	0.87	1.57	0.93	1.63
Yes	0.90	1.58	0.87	1.55	0.93	1.61
Smoking						
TSH						
No	0.14	4.61	0.066	4.418	0.322	4.802
Yes	0.10	3.66	0.026	3.586	0.2822	3.842
FT4						
No	0.80	1.60	0.77	1.57	0.83	1.63
Yes	0.80	1.50	0.77	1.47	0.83	1.53

## References

[1] Velasco I., Sánchez-Gila M., Manzanares S., Taylor P., García-Fuentes E. (2020). Iodine Status, Thyroid Function, and Birthweight: A Complex Relationship in High-Risk Pregnancies. J. Clin. Med..

[2] Vila L., Velasco I., González S., Morales F., Sánchez E., Lailla J.M., Txanton M.-A., Manel P.-D. (2012). Detección de la disfunción tiroidea en la población gestante: Está justificado el cribado universal. Conferencia de consenso. Med. Clin..

[3] Alexander E.K., Pearce E.N., Brent G.A., Brown R.S., Chen H., Dosiou C., Grobman W.A., Laurberg P., Lazarus J.H., Mandel S.J. (2017). Guidelines of the American Thyroid Association for the Diagnosis and Management of Thyroid Disease During Pregnancy and the Postpartum. Thyroid.

[4] Capel I., Corcoy Pla R., Cabrero L., Saldivar D., Cabrillo E. (2010). Enfermedades Tiroideas y Gestación. Obstetricia y Medicina Materno-Fetal.

[5] Endocrine Society, American Association Clinical Endocrinologists, Asia & Oceania Thyroid Association, American Thyroid Association, European Thyroid Association, Latin American Thyroid Association (2007). Management of thyroid dysfunction during pregnancy and postpartum: An Endocrine Society Clinical Practice Guideline. Thyroid.

[6] Bocos-Terraz J., Izquierdo-Álvarez S., Banzalero-Flores J., Álvarez-Lahuerta R., Aznar-Sauca A., Real-López E., Ibáñez-Marco R., Bocanegra-García V., Rivera-Sánchez G. (2009). Thyroid hormones according to gestational age in pregnant Spanish women. BMC Res. Notes..

[7] Vila L., Serra-Prat M., Palomera E., Casamitjana R., De Castro A., Legaz G., Barrionuevo C., Garcia A.-J., Lal-Trehan S., Muñoz J.A. (2010). Reference values for thyroid function tests in pregnant women living in Catalonia, Spain. Thyroid.

[8] García de Guadiana L., González Morales M., Martín-Ondarza González M.C., Martín García E., Martínez Uriarte J., Blázquez Abellán A., Nuevo-García J. (2010). Valoración de la función tiroidea durante la gestación: Intervalos de referencia de tirotropina y tiroxina no unida a proteína durante el primer trimestre. Endocrinol Nutr..

[9] Santiago P., Berrio M., Olmedo P., Velasco I., Sánchez B., García E., Martínez J., Soriguer F. (2011). Reference values for thyroid hormones in the population of pregnant women in Jaen (Spain). Endocrinol Nutr..

[10] Aller Granda J., Rabal Artal A. (2013). Valores de referencia de tirotropina en el primer trimestre del embarazo. Endocrinol Nutr..

[11] Grifol M.L., Gutierrez Menendez M.L., García Menéndez L., Valdazo Revenga M.V. (2013). Valores de referencia y estudio de la variabilidad de hormonas tiroideas en gestantes de El Bierzo. Endocrinol Nutr..

[12] Santiago Fernández P., González-Romero S., Martín Hernández T., Navarro González E., Velasco López I., Millón Ramírez M.C. (2015). Abordaje del manejo de la disfunción tiroidea en la gestación. Documento de consenso de la Sociedad Andaluza de Endocrinología y Nutrición (SAEN). Semergen–Med. de. fam..

[13] Pombar-Pérez M., Penín-Álvarez M., Vélez-Castillo M., Trigo-Barros C., Álvarez-García E., Rodríguez-Ferro R. (2013). Impacto de la aplicación de los criterios de la Asociación Americana de tiroides en el diagnóstico de hipotiroidismo en gestantes de Vigo, España. Rev. Peru. Med. Exp. Salud. Pública..

[14] Diaz-Cadórniga F.J., Delgado-Álvarez E. (2004). Déficit de Yodo en España. Endocrinol Nutr..

[15] Fundación Española de Dietistas y Nutricionistas. Revisión Científica sobre la alimentación en la mujer embarazada. Evidencia Científica. Pamplona: Centro de Análisis de Evidencia Científica (CAEC-FEDN), Consejo General de Dietistas-Nutricionistas: Author. 2014. http://www.fedn.es/docs/alimentacionyembarazoFEDN.pdf.

[16] Wu T., Liu G.J., Li P., Clar C. (2002). Iodized salt for preventing iodine deficiency disorders. CDSR.

[17] De Luís D.A., Aller R., Izaola O. (2005). Problemática de la deficiencia de yodo durante la gestación. Ann. Med. Interna..

[18] Suárez Rodríguez M., Azcona San Julián C., Alzina de Aguilar V. (2013). Ingesta de yodo durante el embarazo: Efectos en la función tiroidea materna y neonatal. Endocrinol. Nutr..

[19] Soriguer F., García-Fuentes E., Gutiérrez-Repiso C., Rojo-Martínez G., Velasco I., Goday A. (2012). Iodine intake in the adult population. Di@bet.es study. Clin. Nutr..

[20] Dunn J.T., Crutchfield H.E., Gutekunst R., Dunn A.D. (1993). Two simple methods for measuring iodine in urine. Thyroid.

[21] Reed A.H., Henry R.J., Mason W.B. (1971). Influence of statistical method used on the resulting estimate of normal range. Clin. Chem..

[22] Karakosta P., Chatzi L., Bagkeris E., Daraki V., Alegakis D., Castanas E., Manolis K., Marilena K. (2011). First and second-trimester reference intervals for thyroid hormones during pregnancy in "Rhea" Mother-Child Cohort, Crete, Greece. J. Thyroid Res.

[23] Lazarus J.H., Bestwick J.P., Channon S., Paradice R., Maina A., Rees R., Chiusano E., John R., Guaraldo V., George L.M. (2012). Antenatal thyroid screening and childhood cognitive function. N. Engl. J. Med..

[24] Amouzegar A., Khazan M., Hedayati M., Azizi F. (2014). An assessment of the iodine status and the correlation between iodine nutrition and thyroid function during pregnancy in an iodine sufficient area. Eur. J. Clin. Nutr..

[25] Soldin O.P. (2006). Thyroid function in pregnancy and thyroid disease: Trimester-specific reference intervals. Therap. Drug. Monit..

[26] International Council For Control of Iodine Deficiency Disorders-ICCIDD (2007). Iodine requirements in pregnancy and infancy. IDD Newsletter..

[27] Saravanan P., Dayan C.M. (2001). Thyroid autoantibodies. Endocrinol. Metab. Clin. North Am..

[28] Plouvier E., Alliot L., Bigorie B., Kowalski C., Medeau V., Thuillier F. (2011). De la nécessité de bien définir les valeurs de référence des hormones thyroïdiennes pour une meilleure interprétation clinique. Ann. Biol. Clin..

[29] Donnay S., Arena J., Lucas A. (2014). Suplementación con yodo durante el embarazo y la lactancia. Toma de posición del Grupo de Trabajo de Trastornos relacionados con la Deficiencia de Yodo y Disfunción Tiroidea de la Sociedad Española de Endocrinología y Nutrición. Endocrinol. Nutr..

[30] Harding K.B., Peña-Rosas J.P., Webster A.C., Yap C.M., Payne B.A., Ota E., De-Regil L.M. (2017). Iodine supplementation for women during the preconception, pregnancy and postpartum period. Cochrane Database Syst. Rev..

[31] Arrizabalaga J.J., Jalón M., Espada M., Cañas M., Latorre P.M. (2015). Concentración de yodo en la leche ultrapasteurizada de vaca. Aplicaciones en la práctica clínica y en la nutrición comunitaria. Med. Clin..

[32] Monckeberg F. (2012). La sal es indispensable para la vida, pero cuánta?. Rev. Chil. Nutr..

[33] Bechtler G., Haeckel R., Horder M., Küffer H., Porth A.J. (1987). Guidelines (1987) for Classification, Calculation and Validation of Conversion Rates in Clinical Chemistry. Clinical Chemistry and Laboratory Medicine.

